# Dental Health Adjuncts and Care: Exploring Access Among Asylum Seekers and Refugees in London, United Kingdom

**DOI:** 10.1177/23800844241293988

**Published:** 2024-11-07

**Authors:** K.J. Hurry, N. Longley, P. Cinardo, H. Chowdhury, A. Ward, S. Eisen

**Affiliations:** 1 Centre for Oral Bioengineering, Institute of Dentistry, Faculty of Medicine and Dentistry, Queen Mary University of London, London, UK; 2 Hospital for Tropical Diseases, University College London Hospitals NHS Foundation Trust, London, UK; 3 London School and Hospital of Tropical Medicine, London, UK; 4 University College London Hospitals NHS Foundation Trust, London, UK; 5 Royal Free London NHS Foundation Trust, London, UK; 6 Central and North West London NHS Foundation Trust, London, UK

**Keywords:** demography, health equity, oral health, dental health services, health-related behavior, displaced persons

## Abstract

**Aims:**

This work examines and describes dental health among people seeking asylum and refugees (PSAR) who are evaluated by the Respond service. This includes access to and use of oral health products, access to dental care, and experience of dental pain.

**Materials and Methods:**

The Respond service pilot offered holistic health assessments to PSAR in temporary accommodation within North Central London between July 2021 and March 2023. Relevant data were extracted from anonymized health records of individuals seen by Respond. Data were analyzed with SPSS (version 28.0.0.0; IBM) to produce descriptive statistics and regression models.

**Results:**

An overall 1,390 PSAR were included; 78.7% were male. The mean ages of adults and children were 31.6 and 6.8 y. Seventy-seven countries of birth were reported, most commonly Iran (23.1%). Over two-thirds (67.1%) of PSAR were not accompanied by family members; only 17.2% had UK family links. The mean travel duration was 769.3 days; migration reasons were multifactorial, including persecution (31.2%) and conflict (20.5%). In addition, 77.3% of PSAR reported having access to a toothbrush; only 50.8% indicated routinely brushing their teeth, with 38.9% having seen a dentist in <36 mo. Dental pain was common (28.8%). Only 45.8% of children (<16 y) had access to a toothbrush, 32.3% were brushing their teeth twice daily, and 9.7% cited dental pain. Logistic regression identified significant predictors of routine toothbrushing, access to dental care, and dental pain. Female PSAR were more likely to routinely brush their teeth (adjusted odds ratio [OR], 3.19; P < 0.001) and access dental care (adjusted OR, 0.57; P < 0.05). PSAR aged 30 to 39 y (adjusted OR, 1.97; P < 0.05) and those with informal travel modes (adjusted OR, 1.82; P < 0.001) were more likely to experience pain.

**Conclusion:**

There is variation in the dental experience of PSAR, but a significant proportion are failing to perform routine toothbrushing, are not regularly accessing dental care, and are experiencing dental pain.

Knowledge Transfer Statement: The results of this analysis suggest that there is variation in the dental experience of people seeking asylum and refugees, but many are failing to perform routine toothbrushing, are not regularly accessing dental care, and are experiencing dental pain.

## Introduction

UK asylum applications have increased over the last 10 y, with 81,130 applications in 2022 (Sturge 2023). On arrival to the United Kingdom, people seeking asylum and refugees (PSAR) are placed in initial accommodation centers and are provided with £47.39 per person per week to spend on food, clothing, and toiletries, including toothbrushes and toothpaste, with additional payments of £3 to £5 per week for pregnant women and children <3 y old (British Red Cross 2023; United Kingdom Government 2023). PSAR placed in “full board” accommodation, where meals are provided, receive just £9.58 per person (United Kingdom Government 2023).

Irrespective of nationality or immigration status, PSAR can register for, access, and receive primary care services from general medical practitioners, dentists, and pharmacies (Office for Health Improvement and Disparities 2023). In England, PSAR and their dependents may be exempt from secondary care charges, including care in hospital or community settings. However, they are expected to pay for NHS dental services like other NHS patients, unless they can provide a valid form of exemption for full or partial dental care costs (e.g., free dental treatment for <18-y-olds).

PSAR are considered a group who experience social and health exclusion (Freeman et al. 2020); they face additional barriers to accessing dental care as compared with their non-PSAR counterparts (Paisi et al. 2020). A recent systematic review highlighted barriers such as affordability, accessibility, and availability of services, as well as limitations of awareness, logistical barriers such as language, and variation in acceptability (Paisi et al. 2020). Many of these barriers can be addressed (e.g., by using an interpreter or providing an asylum support nurse; O’Donnell et al. 2007); however, the scarcity of dental services led to a barrier of access for which a solution could not be easily identified.

Qualitative research has identified that PSAR prioritize safety and security over oral health and, once at a point of stability, the structure of dental systems leads to difficulty in accessing services, which may be compounded by variations in cultural practices and norms (Paisi et al. 2022). The difficulties of meeting the costs of dental care have also been identified as a key barrier (Kang et al. 2019). In particular, Larchanché (2012) found that PSAR who have fled due to persecution were fearful of seeking help and support from health services due to their previous traumatic experiences or through fear of being deported.

PSAR experience extreme oral health inequalities with high levels of oral disease (Keboa et al. 2016; Public Health England 2021a). A scoping review that consisted of studies from 14 countries, including the United Kingdom, found consistently higher rates of caries, periodontal disease, oral lesions, and traumatic dental injury in the PSAR population as compared with national surveys, with 80% of recent immigrants and Bhutanese refugees in Canada having untreated caries and/or periodontal disease (Keboa et al. 2016).

To address this oral disease burden and barriers to access, the UK Health Security Agency has created a 7-step model of care for NHS dentistry that can be used to guide PSAR who require access to dental care and health care professionals (Public Health England 2021b). It focuses on awareness of entitlement to treatment, provides help in finding a dentist, and highlights that translation services are available. The model emphasizes prevention and providing culturally sensitive and person-centered care. However, at the time of writing, we are not aware of any national pathways to support PSAR accessing NHS dental treatment.

Inclusion health services aim to meet the needs of people who are socially excluded, experience overlapping risk factors for poor health (e.g., poverty, violence, and complex trauma), and are subjected to stigma and discrimination such as PSAR (Public Health England 2021a). These groups are often inconsistently accounted for in electronic records (e.g., health care databases), suggesting that our understanding of the burden of oral disease of this cohort is likely only the tip of the iceberg.

The Respond pilot based at University College London Hospital offered holistic health assessments to PSAR placed in initial accommodation centers across the North Central London boroughs of Camden, Barnet, and Islington (Farrant et al. 2022) between July 2021 and March 2023. Patients are assessed by a trained health care professional for physical and mental health needs, as well as social and safeguarding issues, and appropriately signposted to relevant services before care planning and onward referral. These assessments include questions about dental health, such as access to and use of oral health adjuncts (e.g., toothbrushes and toothpaste), access to dental care, and whether the individual has dental pain, which allows the health care professional to signpost and refer appropriately. All patients are offered the use of a telephone interpreter if required.

Although existing literature focuses on the rates of dental decay in PSAR (Keboa et al. 2016), little has been published about the access that this population has to oral health adjuncts and dental services or the prevalence of self-reported dental pain. No research to date considers dental health in the context of demographic, family, and migration factors.

This work aims to examine and describe dental health among PSAR who are evaluated by the Respond service. This includes access to and use of oral health products, access to dental care, and experiences of dental pain.

## Materials and Methods

A retrospective service evaluation was completed of anonymized patient records of all PSAR assessed by Respond from July 2021 to March 2023; no individuals were excluded. The project was registered as a service evaluation at University College London Hospital; ethical approval was not required.

Routinely collected data were extracted under the themes of demographics, family, migration, and dental. Under the heading of “demographic,” the participant’s age, sex, country of birth, and ethnicity were recorded; accompanying family members and preexisting links to the United Kingdom were recorded under “family.” “Migration” data included the reason for migration, travel duration, and formal versus informal route of migration. “Formal” routes were defined as situations where a visa was granted (e.g., plane travel), including cases where tourist visas were initially given and then asylum claimed; “informal” routes were any other route (e.g., boat travel). Dental data included access to a toothbrush, routine toothbrushing (i.e., twice daily), last visit to the dentist, and dental pain.

The data were sorted in an encrypted and password-protected Excel database. Age was grouped into 4 cohorts with similar frequencies, and dental attendance was dichotomized around 36 mo for descriptive analysis. The data were analyzed with SPSS (version 28.0.0.0; IBM) to report descriptive statistics. A conceptual map was used to identify which variables should be included in the analysis (available on request). Cross-tabulations and logistic regression models were used to identify associations between PSAR characteristics and access to and use of oral health adjuncts, access to dental care, and dental pain. Partial deletion was used in cases of missing data. *P* < 0.05 was chosen as the threshold to declare statistical significance.

## Results

### Demographics

In total, 1,390 PSAR were assessed by Respond from July 2021 to March 2023; population characteristics are presented in Table 1. The majority were male (*n* = 1,094, 78.7%), and the mean age overall was 28.9 y (range, 0 to 77). The mean age was 31.6 y for the adult cohort (>16 y) and 6.8 y for the child cohort.

**Table 1.** PSAR Population Characteristics and Family and Migration Data (*N* = 1,390).

	No. (%)
Age, y	
<9	111 (8.0)
10 to 19	78 (5.6)
20 to 29	589 (42.4)
30 to 39	392 (28.2)
40 to 49	156 (11.2)
50 to 59	45 (3.2)
≥60	19 (1.4)
Sex	
Male	1,094 (78.7)
Female	295 (21.2)
Not disclosed	1 (0.1)
Accompanying family	
Yes	384 (27.6)
No	933 (67.1)
NA	73 (5.3)
Family links in the UK	
Yes	239 (17.2)
No	964 (69.4)
NA	187 (13.5)
Travel mode	
Formal	318 (22.9)
Informal	907 (65.3)
NA	165 (11.9)

NA, not available; PSAR, people seeking asylum and refugees.

PSAR originated from 77 countries of birth, as shown in the Figure, most frequently Iran (*n* = 321, 23.1%); for 4.8% of participants, no country of origin was documented. Of the 23 children <2 y old, 21 (91.3%) were born in the United Kingdom.

**Figure.**
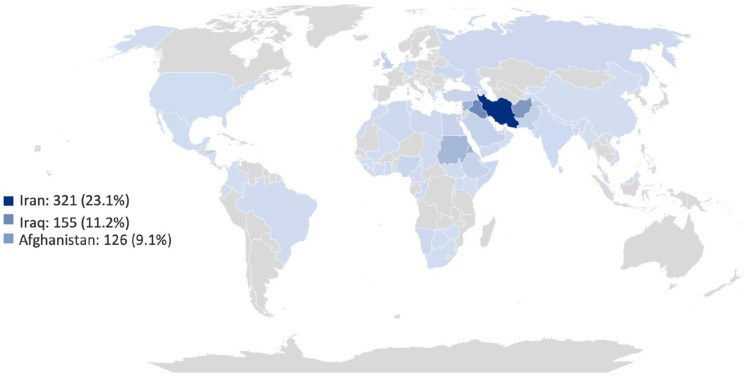
Country of birth of population. Image shows the highlighted areas of countries of birth given by the PSAR population, with a key detailing the 3 most reported countries of birth including the number and percentage of PSAR. PSAR, people seeking asylum and refugees.

### Family

Most individuals (*n* = 933, 67.1%) were lone migrants (i.e., did not report having an accompanying family member); all children (≤16 y) were accompanied by a parent. In total, 964 (69.4%) had no family links within the United Kingdom. Those adult PSAR who did have family links tended to be distant relatives, such as cousins, aunts, and uncles, based outside of London.

### Migration

The mean travel duration was 769.3 d (range, 0 to 15,706). Most PSAR arrived in the United Kingdom through an informal travel route (*n* = 907, 65.3%). Reasons for migration were varied and multifactorial: persecution (*n* = 434, 31.2%), conflict (*n* = 285, 20.5%), torture (*n* = 18, 1.3%), modern slavery (*n* = 7, 0.5%), female genital mutilation (*n* = 6, 0.4%), sexual violence (*n* = 3, 0.2%), and famine (*n* = 1, 0.1%).

### Dental Factors

Between 12.5% to 27.0% of PSAR chose not to respond to dental questions (Table 2). Most PSAR reported having access to a toothbrush (77.3%, *n* = 1,074), but only 50.8% indicated routinely brushing their teeth (*n* = 706).

**Table 2.** PSAR Population Dental Data (*N* = 1,390).

	No. (%)
Access to a toothbrush	
Yes	1,074 (77.3)
No	53 (3.8)
NA	263 (18.9)
Routinely brushing teeth	
Yes	706 (50.8)
No	339 (24.4)
NA	345 (24.8)
Time since last dental appointment, mo	
0 to 6	186 (13.4)
>6 to 12	123 (8.8)
>12 to 24	135 (9.7)
>24 to 36	97 (7.0)
>36	474 (34.1)
NA	375 (27.0)
Dental pain	
Yes	401 (28.8)
No	815 (58.6)
NA	174 (12.5)

NA, not available; PSAR, people seeking asylum and refugees.

Only 38.9% (*n* = 541) of PSAR reported having seen a dentist in the previous 36 mo. In the adult cohort (>16 y), 32.7% (*n* = 404) of PSAR had seen a dentist in the previous 24 mo in line with NICE recall guidelines (National Institute for Health and Care Excellence 2004). Many PSAR cited dental pain (*n* = 401, 28.8%).

Subanalysis of the child cohort (≤16 y) showed that few had access to a toothbrush (*n* = 71, 45.8%), and even fewer were routinely brushing their teeth twice per day (*n* = 50, 32.3%). Furthermore, only 21.3% (*n* = 33) had seen a dentist in the previous 12 mo, and 9.7% (*n* = 15) were in pain due to their teeth.

Statistical analysis demonstrated that PSAR in the 30- to 39-y-old group more commonly owned a toothbrush than other age groups (*P* < 0.001; Table 3). Being aged ≥40 y (*P* = 0.003), female sex (*P* < 0.001), having accompanying family members (*P* < 0.001), and arriving through formal routes (*P* = 0.01) were all associated with higher rates of regular brushing. Being aged ≥40 y (*P* = 0.04), female sex (*P* = 0.002), and having accompanying family members (*P* = 0.02) were associated with having accessed dental care in <36 mo. Being aged 30 to 39 y (*P* = 0.006), having no accompanying family (*P* = 0.02), having no family links in the United Kingdom (*P* = 0.03), and arriving via informal routes (*P* = 0.01) were all associated with higher rates of dental pain.

**Table 3.** Cross-tabulation of Demographic, Family, and Migratory Factors with Dental Factors.

	Access to Toothbrush		Routine Toothbrushing		Access to Dental Care		Dental Pain	
	Yes, %	*P* Value	Yes, %	*P* Value	Yes, %	*P* Value	Yes, %	*P* Value
Age, y		<0.001		0.003		0.04		0.006
<20	86.8		69.1		57.1		20.2	
20 to 29	95.5		62.8		48.2		33.9	
30 to 39	97.5		68.4		57.2		37.0	
≥40	96.2		78.0		57.7		31.6	
Sex		0.87		<0.001		0.002		0.17
Male	95.2		63.0		51.0		33.9	
Female	95.5		86.6		63.7		29.2	
Accompanying family		0.47		<0.001		0.02		0.02
Yes	94.4		79.3		59.3		27.6	
No	95.5		63.5		51.0		35.2	
Family links in the UK		0.21		0.07		0.14		0.03
Yes	97.1		73.1		58.0		26.2	
No	95.1		66.3		52.1		34.1	
Travel mode		0.62		0.01		0.45		0.01
Formal	96.6		75.8		55.6		25.7	
Informal	96.0		64.7		52.8		35.8	

There were no statistically significant associations when analysing dental pain compared to having a toothbrush, routinely brushing or visiting the dentist regularly.

Logistic regression analysis identified significant predictors of PSAR having access to a toothbrush, routinely toothbrushing, accessing dental care, and having dental pain (Table 4). Adjusting for covariates in the final adjusted model identified only 3 significant independent predictors of these dental factors: sex, age, and travel mode. Female PSAR were more likely than male PSAR to routinely brush their teeth (adjusted odds ratio [OR], 3.19; *P* < 0.001) and access dental care (adjusted OR, 0.57; *P* < 0.05). PSAR in the 30- to 39-y-old group were more likely than their counterparts to experience dental pain (adjusted OR, 1.97; *P* < 0.05). Those PSAR who entered the United Kingdom through informal routes were more likely to experience pain (adjusted OR, 1.82; *P* < 0.001) than those who used formal routes.

**Table 4.** Logistic Regression Analysis of the Demographic, Family, and Migratory Factors with Dental Factors.

	Access to Toothbrush		Routine Toothbrushing		Access to Dental Care		Dental Pain	
Characteristic	Unadjusted	Adjusted	Unadjusted	Adjusted	Unadjusted	Adjusted	Unadjusted	Adjusted
Age, y								
<20	Reference	Reference	Reference		Reference		Reference	Reference
20 to 29	**3.12 (1.60, 6.29)** ^**^	1.01 (0.26, 3.92)	0.76 (0.47, 1.22)		1.43 (0.91, 2.25)		**2.04 (1.27, 3.24)** ^*^	1.56 (0.88, 2.74)
30 to 39	**6.00 (2.46, 14.53)** ^**^	2.08 (0.47, 9.08)	0.97 (0.59, 1.59)		1.00 (0.62, 1.60)		**2.32 (1.44, 3.76)** ^^**^^	**1.97 (1.10, 3.51)** ^*^
≥40	**3.85 (1.52, 9.77)** ^*^	1.02 (0.24, 4.37)	1.59 (0.90, 2.79)		0.98 (0.58, 1.64)		**1.83 (1.08, 3.10)** ^*^	1.64 (0.89, 3.03)
Sex								
Male	Reference		Reference	Reference	Reference	Reference	Reference	
Female	1.06 (0.52, 2.14)		**3.79 (2.47, 5.81)** ^^**^^	**3.19 (1.86, 5.49)** ^^**^^	**0.59 (0.43, 0.82)** ^*^	**0.57 (0.37, 0.88)** ^*^	0.80 (0.59, 1.10)	
Accompanying family								
Yes	Reference		Reference	Reference	Reference	Reference	Reference	Reference
No	1.25 (0.69, 2.29)		**0.45 (0.33, 0.63)** ^^**^^	0.72 (0.45, 1.15)	**1.40 (1.04, 1.87)** ^*^	0.99 (0.65, 1.51)	**1.43 (1.07, 1.90)** ^*^	0.95 (0.62, 1.44)
Family links in the UK								
Yes	Reference		Reference		Reference		Reference	Reference
No	0.58 (0.24, 1.37)		0.73 (0.51, 1.03)		1.27 (0.92, 1.75)		**1.46 (1.05, 2.02)** ^*^	1.37 (0.96, 1.95)
Travel mode								
Formal	Reference		Reference	Reference	Reference		Reference	Reference
Informal	0.82 (0.39, 1.76)		**0.59 (0.42, 0.81)** ^*^	0.80 (0.56, 1.15)	1.12 (0.84, 1.49)		**1.62 (1.20, 2.18)** ^*^	**1.82 (1.30, 2.55)** ^^**^^

Data are presented as odds ratio (95% CI). The adjusted model includes only those variables that were statistically significant (*P* < 0.05) in the unadjusted (bivariate) analysis.

* *P* < 0.05. ^**^P < 0.001.

Owing to variations in and incomplete collection of data, country of birth and ethnicity could not be included in the analysis.

## Discussion

We present data regarding dental health for a cohort of 1,390 PSAR. We have shown that although the majority had access to a toothbrush, just over half were using it regularly, less than two-fifths had seen a dentist in the preceding 36 mo, and more than a quarter had dental pain.

Our cohort reported a high frequency of toothbrush ownership (*n* = 1,074, 77.3%). This is perhaps higher than expected, given that toothbrushes are not routinely provided to PSAR on arrival in the United Kingdom; instead, PSAR are expected to purchase a toothbrush with their weekly allowance, which also covers food, sanitation, and clothing (United Kingdom Government 2023). In initial accommodation centers, PSAR may be provided with a “welcome pack,” which may sometimes include oral health adjuncts such as a toothbrush and toothpaste, thus possibly explaining the high proportion of toothbrush ownership among PSAR; children may also be provided with a toothbrush at school and play centers. While data on the percentage of the UK population who own a toothbrush could not be identified, there are reports that in 2020, 34 million (67%) UK adults owned an electric toothbrush, suggesting that total rates of toothbrush ownership are likely to be higher than in our PSAR population (Oral Health Foundation 2020).

Despite most PSAR owning a toothbrush, just over half (50.8%) were routinely brushing their teeth, as compared with 71% of UK adults who report twice-daily brushing (Rutland and Mackenzie 2022). This is consistent with lower rates of routine brushing, including once daily, in PSAR populations in a systematic review examining the oral health behaviors of PSAR in Europe (Pabbla et al. 2021). This review also found that parents started brushing their children’s teeth at an older age and struggled to find time to supervise toothbrushing; this may explain reduced rates of toothbrush access (*n* = 71, 45.8%) and routine brushing (*n* = 50, 32.3%) in our child cohort. Although PSAR may be provided with a toothbrush, they may not be provided with adequate oral health education on how to use it, and because oral health is a lower priority at a time of stress, uncertainty, and instability, rates of twice-daily brushing are substantially lower. This may represent a missed opportunity to improve dental care in this population by providing basic education.

Cultural differences in oral health care should be acknowledged. Although not recorded in this study, miswak—a traditional tooth-cleaning twig from the *Salvadora persica* tree—is used instead of toothbrushes throughout Asia, Africa, South America, and the Middle East, meaning that some PSAR may not relate to questions about routine toothbrushing (Haque and Alsareii 2015). PSAR who had accompanying family and those who used formal routes were associated with higher rates of routine brushing, although this was not statistically significant in adjusted regression analysis. This may be due to a sense of safety and security resulting from the existence of some social and community support and the greater certainty about the future provided by arrival via formal routes, enabling people to prioritize their dental health (Paisi et al. 2022).

NICE guidance recommends a maximum interval of 24 mo between oral health reviews for adults and 12 mo for children (National Institute for Health and Care Excellence 2004). Over one-third of PSAR had not seen a dentist in the preceding 36 mo (*n* = 541, 38.9%). Between the adult and child PSAR cohorts, 32.7% of adults had seen a dentist in the previous 24 mo and 21.3% of children in the previous 12 mo. This finding is lower than that of the annual report of NHS Dental Statistics in June 2022, which noted that 36.9% of adults had seen a dentist in the previous 24 mo and 46.2% of children in the previous 12 mo (NHS Dental Statistics 2022). Our findings are analogous to a recent systematic review of migrant health care utilization, which found that “host populations” were more accustomed to regular dental attendance whereas PSAR populations were more likely to attend for emergency treatment (Pabbla et al. 2021). This may reflect a need to prioritize safety and survival over such issues as dental health (Paisi et al. 2022).

It should be noted that our cohort was universally placed in initial accommodation centers, thus likely early in their asylum claim and at a time of significant instability. The majority did not have preexisting links to the United Kingdom or accompanying family members. These are postmigratory factors that are recognized to pose substantial mental health impacts because of the “gradual fragmentation of identity, belonging and perceptions around one’s social value,” precarious living conditions, and feelings of social isolation (Rowley et al. 2020; Trueba et al. 2023). In our cohort, having accompanying family meant that PSAR were more likely to have seen a dentist, perhaps suggesting that, in the context of greater social support, prioritization of dental health becomes more feasible. The markedly lower rate of timely engagement with dental services in PSAR, particularly for children, is likely to contribute to the oral health inequalities in this population (Public Health England 2021a).

Female PSAR were more likely than male PSAR to routinely brush their teeth (adjusted OR, 3.19; *P* < 0.001) and access dental care (adjusted OR, 0.57; *P* < 0.05). Similar results have been identified at a national level in the United Kingdom (Office for Health Improvement and Disparities 2024). The 2021 Adult Oral Health Survey found that women were more likely than men to brush twice per day (82% vs. 72%) and attend the dentist for regular check-ups (68% vs. 57%). There is also some suggestion that females settle better into United Kingdom–based standards of dental health (Paisi et al. 2022). Further qualitative analysis into this “successful” cohort may be valuable to stakeholders when planning pathways to dental care for PSAR.

Over one-quarter of the PSAR reported dental pain, which was lower than that found in comparable audits and service evaluations on the PSAR population (Keboa et al. 2016). It is possible that subjective rates of dental pain were lower in our cohort, which was significantly biased toward males, due to gender-based cultural expectations around the perception of expression of pain as indicative of weakness (Peacock and Patel 2008; Gautrau 2021). However, the rates of dental pain were markedly higher than the rates in the 2021 Adult Oral Health Survey, where 10% of adults with teeth, fillings, crowns, or fixed bridges self-reported dental pain (Office for Health Improvement and Disparities 2024). In the context of the high prevalence of dental pain, these low rates of routine toothbrushing and dental attendance are of particular concern.

Ages 30 to 39 y and entering the United Kingdom through informal routes were predictors of dental pain in adjusted logistical regression analysis. This possible impact of age on dental pain aligns with the 2021 Adult Oral Health Survey where dental pain was most commonly reported in adults aged 35 to 44 y (13% of adults; Office for Health Improvement and Disparities 2024). With regard to the route of travel, this may again reflect that those traveling through informal routes are, by necessity, focused on their safety and survival on their journey to and arrival in the United Kingdom and perhaps less focused on preventing dental trauma, dental decay, and gum disease (known causes of dental pain). This relationship could be mediated by a lack of access to dental care during long and unpredictable journeys. Further information regarding the causes of dental pain would be valuable to understand the treatment required and aid dental health resource planning, and research into the reason for these trends could help uncover areas for targeted oral health interventions.

Overall, our data revealed low rates of routine brushing and engagement with routine dental care, coupled with a high prevalence of dental pain, suggesting a lack of awareness of the UK dental system; the provision of targeted oral health promotion and education could be used to overcome this.

In the United Kingdom, <18-y-olds are exempt from paying for NHS dental care and may receive oral hygiene education and fluoride varnish application while in schools and nurseries. However, PSAR may not have come across this in previous host countries and could benefit from support to reintegrate brushing and regular dental attendance as part of their new “normal” (Office for Health Improvement and Disparities 2023).

The use of community-led oral health promotion programs for humanitarian migrants has been recommended and implemented regionally in the United States and Australia but is yet to be evaluated (Geltman et al. 2014; Gibbs et al. 2015; Eslamiamirabadi et al. 2022). Evaluation of “culturally appropriate” community-based oral health education interventions was shown to improve knowledge about toothbrushing and attitudes toward toothbrushing and “sugar snacking” in migrant Chinese mothers in Northern Ireland; this also improved the quality of relationship between patient and child (Yuan 2019). A cross-sectional observational study of immigrants in Belgium found a reduction in missed dental appointments through the implementation of a personal assistance program led by community health workers (Lambert 2018). Furthermore, targeted supervised brushing programs and provision of toothbrushes and toothpaste by post and by health visitors in the United Kingdom have resulted in a 3- and 7-times return on investment over a 10-y period, respectively (Public Health England 2016). Although these models are generated from different population groups, these targeted interventions could be considered to improve routine toothbrushing and dental attendance in the PSAR population.

Tackling dental health inequalities through cross–health care collaboration is key to improving oral health outcomes. By involving individuals with lived experience in service evaluation and development, commissioners can be more aware of barriers to accessing health care and cocreate functional pathways accessible to the populations who use them. The inclusion of oral health frameworks to help people marginalized by social exclusion has also been recommended and can assist commissioners in future planning, as implemented in the aforementioned examples (Freeman et al. 2020).

The Respond service currently provides ad hoc dental health education, screening, and treatment by dental care professionals for PSAR children through collaboration with a third sector organization, the Dental Wellness Trust, as well as onward referral to appropriate NHS services (Hurry 2023). This provision of dental care and education is locally based and yet to be evaluated but is likely to represent an effective model of oral health promotion and dental screening to benefit an underserved population.

### Limitations

This study is limited as it included only PSAR in initial accommodation centers within North Central London. A broader population, such as PSAR who have been in the United Kingdom for longer periods and are located outside of London, could increase generalizability. However, the demographics of the Respond cohort are broadly consistent with national statistics for 2021 (United Kingdom Government 2022), in which two-thirds (67%) of asylum applicants were males aged 18 to 49 y (vs. 69.8% in our cohort, *n* = 970), and are representative of national trends and geopolitical events, including increased migration from Eastern Europe.

There are established limitations of self-reported data in the literature: answers may be exaggerated; participants may be too embarrassed to share private information; and the answers are susceptive to various biases (Northrup 1997).

Another limitation is that the dental questions were part of a much broader health assessment with little dental focus and the responses to dental questions were not documented for between 12.5% and 27.6% of participants. These missing data may have affected the results. The results could be improved by using a more in-depth questionnaire regarding dental health and cocreation of qualitative work with PSAR to reduce result bias and explore these factors affecting the use of oral health adjuncts and access to dental care.

### Further Research

Further research into risk factors for poor oral health in vulnerable populations is needed to develop an evidence base to underpin interventions such as oral health promotion and prioritized dental treatment and to supplement our knowledge with clinical data on normative need. The inclusion of PSAR within national epidemiologic surveys is necessary to understand the full extent of their access to oral adjuncts and dental care and the prevalence of dental pain. The development of pictorial educational materials and training of peers to deliver oral health education could be used to improve PSAR adherence to United Kingdom–based optimal oral care.

Regional dental care pathways should be evaluated and streamlined to develop a national pathway for PSAR for routine and emergency dentistry, and such pathways should take into consideration high-risk characteristics and known barriers to accessing dental care by utilizing, for example, on-site pop-up dental clinics (Paisi et al. 2020).

## Conclusion

The PSAR screened by Respond experienced a range of access to oral health adjuncts and dental care. Despite owning toothbrushes, many individuals are failing to perform routine toothbrushing, are not routinely accessing dental care, and are reporting dental pain. Age, sex, and mode of migration were all significant predictors in the analysis of access to toothbrushes, routine toothbrushing, dental attendance, and self-reported dental pain.

Integrated inclusion health care systems can aid this excluded population in accessing dental care by utilizing cross–health care collaboration. The development of a cocreated national pathway, which tackles recognized barriers, would be beneficial in improving routine and emergency access to dental care and promoting the prevention of dental caries through oral health education.

## Author Contributions

K.J. Hurry, contributed to conception and design, contributed to analysis, drafted the manuscript; N. Longley, A. Ward, S. Eisen, contributed to conception and design, critically revised the manuscript; P. Cinardo, H. Chowdhury, contributed to analysis, critically revised the manuscript. All authors have their final approval and agree to be accountable for all aspects of work.
